# Supplementary material for: Modeling glioblastoma heterogeneity as a dynamic network of cell states
Source: Mol Syst Biol. 2021 Sep 16;17(9):e10105. doi: 10.15252/msb.202010105 (PMC8444284; doi:10.15252/msb.202010105)
Supplement: Supplementary file 5 — Source Data for Figure 3 [file MSB-17-e10105-s001.zip › Figure3A_sourcedata/GSEA_3065/nosigdb_state4.GseaPreranked.1622718430994/gsea_report_for_na_neg_1622718430994.html]

Report for na\_neg 1622718430994 [GSEA]

| GS  follow link to MSigDB | GS DETAILS | SIZE | ES | NES | NOM p-val | FDR q-val | FWER p-val | RANK AT MAX | LEADING EDGE || 1 | DOROSZKO\_U3013MG\_CLUSTER\_2 | Details ... | 200 | -0.85 | -3.35 | 0.000 | 0.000 | 0.000 | 716 | tags=82%, list=11%, signal=90% |
| 2 | FELDMAN\_2020\_G1 | Details ... | 146 | -0.87 | -3.33 | 0.000 | 0.000 | 0.000 | 482 | tags=82%, list=8%, signal=86% |
| 3 | DOROSZKO\_U3180MG\_CLUSTER\_3 | Details ... | 426 | -0.80 | -3.30 | 0.000 | 0.000 | 0.000 | 941 | tags=70%, list=15%, signal=77% |
| 4 | LARSSON\_STATE\_4 | Details ... | 100 | -0.92 | -3.29 | 0.000 | 0.000 | 0.000 | 413 | tags=92%, list=7%, signal=97% |
| 5 | GAROFANO\_IAVARONE\_PPR\_SUBTYPE | Details ... | 328 | -0.79 | -3.22 | 0.000 | 0.000 | 0.000 | 1024 | tags=71%, list=16%, signal=81% |
| 6 | RICHARDS\_ZHONG\_NATURE\_2018\_NPCS\_UPREG | Details ... | 120 | -0.86 | -3.18 | 0.000 | 0.000 | 0.000 | 480 | tags=76%, list=8%, signal=81% |
| 7 | RICHARDS\_NOWAKOWSKI\_SCIENCE\_2017\_MGE\_IPC2\_UPREG | Details ... | 410 | -0.77 | -3.17 | 0.000 | 0.000 | 0.000 | 905 | tags=65%, list=14%, signal=71% |
| 8 | NB\_KI\_CLUSTER5 | Details ... | 85 | -0.88 | -3.10 | 0.000 | 0.000 | 0.000 | 705 | tags=94%, list=11%, signal=105% |
| 9 | SEURAT\_CELLCYCLE | Details ... | 90 | -0.86 | -3.07 | 0.000 | 0.000 | 0.000 | 629 | tags=83%, list=10%, signal=91% |
| 10 | LARSSON\_STATE\_3 | Details ... | 86 | -0.83 | -2.95 | 0.000 | 0.000 | 0.000 | 726 | tags=79%, list=12%, signal=88% |
| 11 | FELDMAN\_2020\_S-G2 | Details ... | 318 | -0.71 | -2.87 | 0.000 | 0.000 | 0.000 | 711 | tags=63%, list=11%, signal=67% |
| 12 | FELDMAN\_2020\_S | Details ... | 145 | -0.75 | -2.82 | 0.000 | 0.000 | 0.000 | 636 | tags=70%, list=10%, signal=76% |
| 13 | DALMO\_BARCODENUMBER\_UNPUB | Details ... | 190 | -0.71 | -2.78 | 0.000 | 0.000 | 0.000 | 429 | tags=52%, list=7%, signal=54% |
| 14 | RICHARDS\_NEFTEL\_CELL\_2019\_G2\_M | Details ... | 43 | -0.88 | -2.75 | 0.000 | 0.000 | 0.000 | 681 | tags=93%, list=11%, signal=104% |
| 15 | SUVA\_G2M\_PMID\_31327527 | Details ... | 43 | -0.88 | -2.72 | 0.000 | 0.000 | 0.000 | 681 | tags=93%, list=11%, signal=104% |
| 16 | FELDMAN\_2020\_G2-M | Details ... | 280 | -0.68 | -2.71 | 0.000 | 0.000 | 0.000 | 810 | tags=61%, list=13%, signal=67% |
| 17 | LARSSON\_STATE\_2 | Details ... | 85 | -0.77 | -2.71 | 0.000 | 0.000 | 0.000 | 1073 | tags=81%, list=17%, signal=97% |
| 18 | RICHARDS\_NEFTEL\_CELL\_2019\_G1\_S | Details ... | 27 | -0.92 | -2.63 | 0.000 | 0.000 | 0.000 | 480 | tags=96%, list=8%, signal=104% |
| 19 | IVY\_CT | Details ... | 138 | -0.70 | -2.63 | 0.000 | 0.000 | 0.000 | 488 | tags=48%, list=8%, signal=51% |
| 20 | SUVA\_G1S\_PMID\_31327527 | Details ... | 27 | -0.92 | -2.61 | 0.000 | 0.000 | 0.000 | 480 | tags=96%, list=8%, signal=104% |
| 21 | LARSSON\_STATE\_1 |  | 97 | -0.69 | -2.48 | 0.000 | 0.000 | 0.000 | 798 | tags=52%, list=13%, signal=58% |
| 22 | FELDMAN\_2020\_LATEG1 |  | 81 | -0.70 | -2.45 | 0.000 | 0.000 | 0.000 | 511 | tags=53%, list=8%, signal=57% |
| 23 | GAROFANO\_IAVARONE\_LIST\_OF\_NEURAL\_PROGENITOR\_MARKER\_GENES |  | 76 | -0.71 | -2.41 | 0.000 | 0.000 | 0.000 | 893 | tags=63%, list=14%, signal=73% |
| 24 | PARADA\_T1\_UP\_26461091 |  | 56 | -0.73 | -2.40 | 0.000 | 0.000 | 0.000 | 670 | tags=54%, list=11%, signal=59% |
| 25 | RICHARDS\_NOWAKOWSKI\_SCIENCE\_2017\_RG\_EARLY\_UPREG |  | 364 | -0.58 | -2.36 | 0.000 | 0.000 | 0.000 | 681 | tags=38%, list=11%, signal=40% |
| 26 | FELDMAN\_2020\_NEURAL G0 |  | 351 | -0.57 | -2.34 | 0.000 | 0.000 | 0.000 | 681 | tags=45%, list=11%, signal=47% |
| 27 | DOROSZKO\_U3054MG\_CLUSTER\_6 |  | 93 | -0.65 | -2.31 | 0.000 | 0.000 | 0.000 | 1543 | tags=71%, list=25%, signal=93% |
| 28 | FELDMAN\_2020\_M-EARLYG1 |  | 103 | -0.63 | -2.28 | 0.000 | 0.000 | 0.000 | 541 | tags=49%, list=9%, signal=52% |
| 29 | RICHARDS\_NOWAKOWSKI\_SCIENCE\_2017\_MGE\_RG2\_UPREG |  | 379 | -0.56 | -2.28 | 0.000 | 0.000 | 0.000 | 676 | tags=37%, list=11%, signal=39% |
| 30 | DOROSZKO\_U3180MG\_CLUSTER\_4 |  | 148 | -0.55 | -2.08 | 0.000 | 0.000 | 0.002 | 1021 | tags=42%, list=16%, signal=49% |
| 31 | PARADA\_T2\_UP\_26461091 |  | 52 | -0.63 | -2.03 | 0.000 | 0.000 | 0.003 | 670 | tags=42%, list=11%, signal=47% |
| 32 | DOROSZKO\_U3180MG\_CLUSTER\_2 |  | 406 | -0.48 | -2.00 | 0.000 | 0.000 | 0.003 | 1666 | tags=48%, list=27%, signal=61% |
| 33 | VANGRONINGEN\_ADRN |  | 182 | -0.49 | -1.88 | 0.000 | 0.001 | 0.030 | 679 | tags=27%, list=11%, signal=29% |
| 34 | ALMSTEDT\_RISK |  | 27 | -0.63 | -1.81 | 0.005 | 0.001 | 0.076 | 679 | tags=41%, list=11%, signal=45% |
| 35 | KLING\_GBM\_PHENO\_PMID\_27667176 |  | 111 | -0.48 | -1.74 | 0.000 | 0.004 | 0.196 | 1285 | tags=39%, list=21%, signal=48% |
| 36 | RICHARDS\_NOWAKOWSKI\_SCIENCE\_2017\_MGE\_IPC3\_UPREG |  | 93 | -0.48 | -1.73 | 0.000 | 0.004 | 0.224 | 698 | tags=31%, list=11%, signal=35% |
| 37 | LARSSON\_STATE\_5 |  | 97 | -0.46 | -1.65 | 0.000 | 0.010 | 0.439 | 726 | tags=42%, list=12%, signal=47% |
| 38 | ALMSTEDT\_VGR\_DIFF |  | 483 | -0.39 | -1.65 | 0.000 | 0.010 | 0.449 | 874 | tags=25%, list=14%, signal=26% |
| 39 | IVY\_CTMVP |  | 37 | -0.54 | -1.64 | 0.007 | 0.010 | 0.453 | 1141 | tags=46%, list=18%, signal=56% |
| 40 | NB\_KI\_CLUSTER8 |  | 223 | -0.39 | -1.55 | 0.001 | 0.022 | 0.745 | 1691 | tags=39%, list=27%, signal=52% |
| 41 | ANGIOGENESIS\_GO\_0001525 |  | 17 | -0.59 | -1.53 | 0.045 | 0.027 | 0.814 | 1109 | tags=35%, list=18%, signal=43% |
| 42 | NB\_KI\_CLUSTER7 |  | 130 | -0.40 | -1.46 | 0.015 | 0.045 | 0.945 | 1783 | tags=40%, list=28%, signal=55% |
| 43 | NB\_KI\_CLUSTER3 |  | 121 | -0.39 | -1.44 | 0.018 | 0.052 | 0.966 | 1397 | tags=40%, list=22%, signal=50% |
| 44 | VANGRONINGEN\_MES |  | 304 | -0.35 | -1.41 | 0.003 | 0.066 | 0.986 | 1215 | tags=31%, list=19%, signal=36% |
| 45 | OLSEN\_ADRENERGIC |  | 63 | -0.42 | -1.40 | 0.045 | 0.070 | 0.989 | 413 | tags=22%, list=7%, signal=24% |
| 46 | NB\_KI\_CLUSTER24 |  | 79 | -0.39 | -1.39 | 0.041 | 0.076 | 0.991 | 1860 | tags=44%, list=30%, signal=62% |
| 47 | KRONA\_ROSEN\_WHITE\_MATTER\_INVASION |  | 130 | -0.37 | -1.37 | 0.042 | 0.083 | 0.995 | 1171 | tags=25%, list=19%, signal=30% |
| 48 | RICHARDS\_NEFTEL\_CELL\_2019\_MES1 |  | 39 | -0.44 | -1.36 | 0.086 | 0.093 | 0.997 | 727 | tags=28%, list=12%, signal=32% |
| 49 | SUVA\_MES1\_PMID\_31327527 |  | 39 | -0.44 | -1.35 | 0.082 | 0.096 | 0.998 | 727 | tags=28%, list=12%, signal=32% |
| 50 | SINGH\_SOX2\_UPREGULATED\_TRANSCRIPTS\_PMID\_28122245 |  | 322 | -0.33 | -1.33 | 0.021 | 0.112 | 1.000 | 913 | tags=18%, list=15%, signal=20% |
| 51 | IVY\_CTPAN |  | 86 | -0.37 | -1.32 | 0.061 | 0.118 | 1.000 | 598 | tags=24%, list=10%, signal=27% |
| 52 | KRONA\_ROSEN\_PERIVASCULAR\_INVASION |  | 29 | -0.44 | -1.28 | 0.162 | 0.145 | 1.000 | 1137 | tags=41%, list=18%, signal=50% |
| 53 | NB\_KI\_CLUSTER22 |  | 82 | -0.37 | -1.28 | 0.117 | 0.146 | 1.000 | 1063 | tags=22%, list=17%, signal=26% |
| 54 | RICHARDS\_LIDDELOW\_NATURE\_2017\_A2\_REACTIVE\_ASTROCYTES |  | 23 | -0.47 | -1.28 | 0.162 | 0.144 | 1.000 | 361 | tags=35%, list=6%, signal=37% |
| 55 | ALMSTEDT\_11Q\_MSIGDB |  | 206 | -0.30 | -1.19 | 0.137 | 0.249 | 1.000 | 1056 | tags=23%, list=17%, signal=27% |
| 56 | GANDAL\_ALCOHOLADDICTION\_DN\_PMID29439242 |  | 51 | -0.36 | -1.16 | 0.237 | 0.292 | 1.000 | 1075 | tags=29%, list=17%, signal=35% |
| 57 | WANG\_PRONEURAL\_PMID\_28697342 |  | 15 | -0.47 | -1.15 | 0.277 | 0.299 | 1.000 | 898 | tags=27%, list=14%, signal=31% |
| 58 | ANASTASSIOU\_MULTICANCER\_INVASIVENESS\_PMID\_22208948 |  | 22 | -0.41 | -1.11 | 0.347 | 0.373 | 1.000 | 393 | tags=14%, list=6%, signal=14% |
| 59 | DALMO\_EXTINCTION\_UNPUB |  | 17 | -0.42 | -1.09 | 0.326 | 0.405 | 1.000 | 1518 | tags=59%, list=24%, signal=77% |
| 60 | NB\_KI\_CLUSTER14 |  | 152 | -0.29 | -1.09 | 0.281 | 0.398 | 1.000 | 1187 | tags=24%, list=19%, signal=29% |
| 61 | NB\_KI\_CLUSTER16 |  | 61 | -0.32 | -1.09 | 0.317 | 0.397 | 1.000 | 1307 | tags=33%, list=21%, signal=41% |
| 62 | WANG\_MESENCHYMAL\_PMID\_28697342 |  | 17 | -0.42 | -1.06 | 0.387 | 0.449 | 1.000 | 1518 | tags=59%, list=24%, signal=77% |
| 63 | GANDAL\_DEPRESSION\_DN\_PMID29439242 |  | 22 | -0.38 | -1.06 | 0.382 | 0.458 | 1.000 | 779 | tags=23%, list=12%, signal=26% |
| 64 | RICHARDS\_VERHAAK\_CANCERCELL\_2010\_MESENCHYMAL |  | 75 | -0.31 | -1.05 | 0.395 | 0.455 | 1.000 | 848 | tags=21%, list=14%, signal=24% |
| 65 | ALMSTEDT\_ALK |  | 35 | -0.33 | -1.01 | 0.440 | 0.551 | 1.000 | 959 | tags=31%, list=15%, signal=37% |
| 66 | WENG\_STEMNESS\_PMID\_30982771 |  | 29 | -0.34 | -0.98 | 0.492 | 0.600 | 1.000 | 949 | tags=34%, list=15%, signal=40% |
| 67 | ALLEN\_115\_OLIGOL4\_6MOBPCOL18A1 |  | 18 | -0.36 | -0.97 | 0.494 | 0.612 | 1.000 | 192 | tags=17%, list=3%, signal=17% |
| 68 | DEPRETER\_LOW\_NB\_RISK |  | 17 | -0.37 | -0.96 | 0.522 | 0.632 | 1.000 | 679 | tags=18%, list=11%, signal=20% |
| 69 | GANDAL\_IBD\_DN\_PMID29439242 |  | 17 | -0.36 | -0.93 | 0.575 | 0.686 | 1.000 | 222 | tags=18%, list=4%, signal=18% |
| 70 | NB\_KI\_CLUSTER12 |  | 29 | -0.33 | -0.93 | 0.560 | 0.678 | 1.000 | 1235 | tags=31%, list=20%, signal=38% |
| 71 | TIISIG\_PMID\_32649888 |  | 20 | -0.34 | -0.92 | 0.574 | 0.688 | 1.000 | 380 | tags=15%, list=6%, signal=16% |
| 72 | RICHARDS\_NOWAKOWSKI\_SCIENCE\_2017\_MICROGLIA\_UPREG |  | 486 | -0.22 | -0.92 | 0.713 | 0.695 | 1.000 | 1031 | tags=18%, list=16%, signal=20% |
| 73 | SINGH\_SOX2\_DOWNREGULATED\_TRANSCRIPTS\_PMID\_28122245 |  | 64 | -0.27 | -0.90 | 0.628 | 0.728 | 1.000 | 509 | tags=13%, list=8%, signal=13% |
| 74 | ALMSTEDT\_17Q\_MSIGDB |  | 246 | -0.21 | -0.82 | 0.878 | 0.859 | 1.000 | 520 | tags=10%, list=8%, signal=10% |
| 75 | RICHARDS\_NOWAKOWSKI\_SCIENCE\_2017\_IN\_CTX\_CGE2\_UPREG |  | 174 | -0.21 | -0.79 | 0.881 | 0.890 | 1.000 | 1605 | tags=31%, list=26%, signal=41% |
| 76 | NB\_KI\_CLUSTER15 |  | 87 | -0.22 | -0.78 | 0.859 | 0.903 | 1.000 | 1457 | tags=21%, list=23%, signal=27% |
| 77 | ALLEN\_114\_OPCL1\_6MYT1 |  | 16 | -0.29 | -0.76 | 0.768 | 0.917 | 1.000 | 1753 | tags=44%, list=28%, signal=61% |
| 78 | PARADA\_T1\_DN\_26461091 |  | 30 | -0.23 | -0.67 | 0.914 | 0.973 | 1.000 | 376 | tags=7%, list=6%, signal=7% |
| 79 | NB\_KI\_CLUSTER6 |  | 304 | -0.16 | -0.64 | 0.999 | 0.972 | 1.000 | 1720 | tags=26%, list=27%, signal=34% |
Table: Gene sets enriched in phenotype **na **[plain text format]****
